# Metal Oxides Catalyze Formation of Dehydroamino Acids by Phosphate β‐Elimination Under Physiological Conditions

**DOI:** 10.1002/anie.9805066

**Published:** 2026-09-01

**Authors:** Samuel W. Markovich, Brian L. Frey, Mark Scalf, Lloyd M. Smith

**Affiliations:** ^1^ Chemistry University of Wisconsin – Madison University Ave Madison Wisconsin USA

**Keywords:** alzheimer's disease, dehydroamino acids, goethite, hematite, posttranslational modification

## Abstract

The dehydroamino acids (DHAAs) dehydroalanine and dehydrobutyrine are formed in proteins posttranslationally from serine and threonine, respectively. They contain an electrophilic alkene, which reacts readily with nucleophiles by Michael addition, giving rise to a variety of chemical modifications as well as protein crosslinks. Recent work has shown DHAAs and their modifications to be highly prevalent in protein aggregates isolated from brains of Alzheimer's Disease (AD) patients. As there is no human enzyme known to catalyze the formation of DHAAs, this raised the question of how they are produced. We show here that metal oxides, particularly the iron oxides goethite and hematite, efficiently catalyze the formation of DHAAs in model phosphopeptides in vitro under physiologically relevant conditions. Iron oxides are known to accumulate in the human brain with age, and this deposition is enhanced in neurodegenerative disorders like AD. The DHAA formation by heterogeneous catalysis reported here provides a previously unknown mechanism linking iron deposition to protein crosslinking in AD and potentially other neurodegenerative diseases where iron accumulation and protein aggregation co‐occur.

## Graphical abstract

Dehydroamino acids (DHAA) are atypical electrophilic sites that lead to crosslinked proteins in Alzheimer's disease (AD) brains. No catalyst for their formation has been known in humans. We report that metal oxides catalyze β‐elimination of phosphoserine and phosphothreonine in model peptides at pH 7.5°C and 37°C, yielding DHAAs. This represents a potential mechanism for DHAA formation in humans.

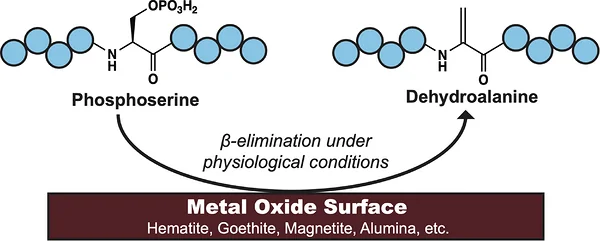

## 1 Introduction

Dehydroamino acids (DHAAs) are electrophilic posttranslational modifications formed through β‐elimination of serine and threonine residues, yielding dehydroalanine and dehydrobutyrine, respectively (Scheme 1) [1, 2]. Phosphorylated forms of these residues are common intermediates for DHAAs both in vitro and in vivo, though other precursors exist [2, 3]. DHAAs contain a characteristic alkene conjugated with the peptide or protein backbone, which functions as a Michael acceptor. This represents a rare example of an electrophilic moiety in a protein, which may readily react with a variety of nucleophiles, including thiols, amines, and phosphines [1].

**SCHEME 1:**
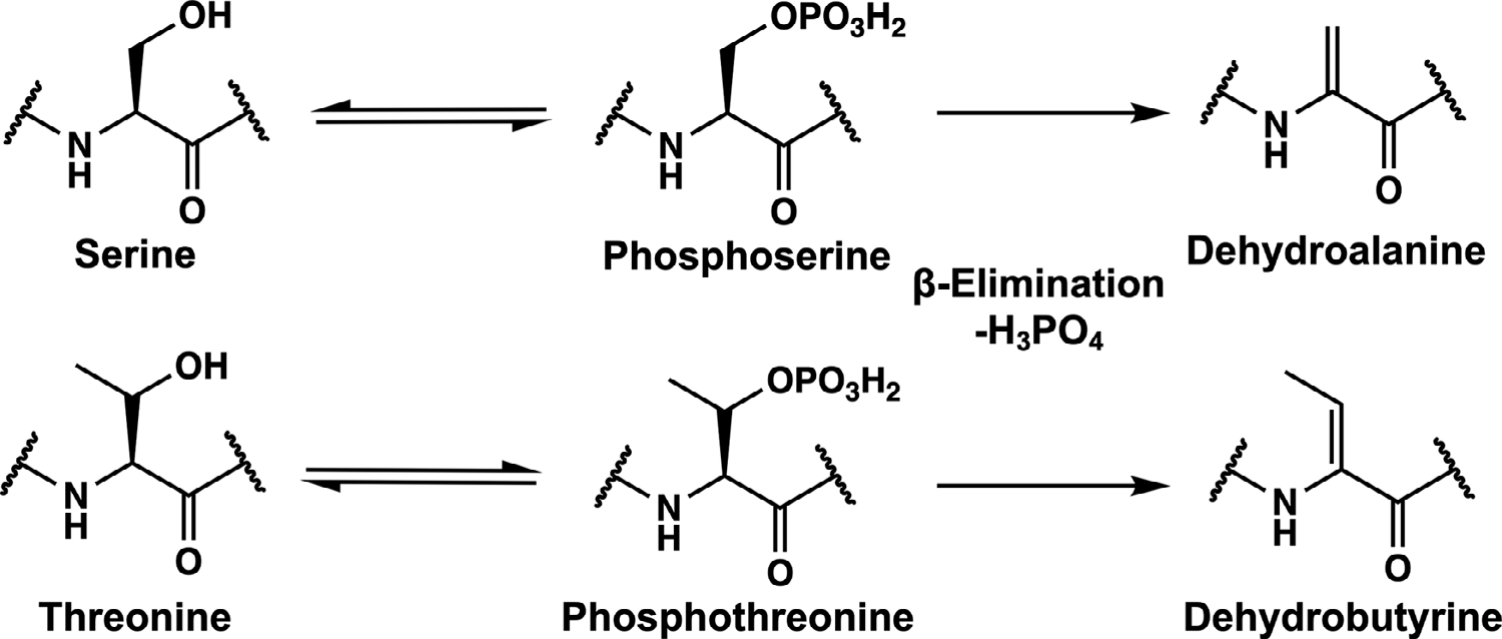
Phosphoserine and phosphothreonine undergo β‐elimination of the phosphate to form dehydroalanine and dehydrobutyrine, respectively.

This reactivity has been exploited in synthetic biochemistry for chemical modification. One of the earliest approaches used Ba(OH)_2_ to generate dehydroalanine from phosphoserine in model peptides, however, these strongly basic conditions (pH > 12) can damage proteins [4, 5]. 2,5‐dibromohexanediamide (DBHDA) has been used for the bisalkylation of cysteine thiols, promoting elimination to DHA [6]. These and other known synthetic strategies [1, 7, 8, 9, 10] are incompatible with physiological conditions.

Enzymatic installation of DHAAs is a well‐characterized feature of prokaryotic peptide biosynthesis. In the context of human biology, however, these enzymes are primarily associated with bacterial pathogenesis; for instance, phosphothreonine lyases generate DHB residues in host kinases to suppress immune signaling [3]. Currently, there are no known endogenous human enzymes capable of generating these residues. Notably, DHAAs have been detected in human proteins within the context of protein aggregation, including in protein aggregates from Alzheimer's disease (AD) brains and age‐related cataracts formed from αB‐crystallin [11, 12, 13, 14]. DHAAs have been shown to induce protein‐protein crosslinking with nucleophilic side chains, such as those of lysine, histidine, or cysteine, potentially contributing to aggregation [11, 13]. DHAA formation in the lens is often attributed to cumulative protein damage, as αB‐crystallin is long‐lived and exposed to ultraviolet (UV) radiation that might promote the gradual formation of DHAAs over time [12, 13]. DHAA formation in AD brains may proceed through a different pathway, as these proteins are regularly turned over and are not exposed to UV radiation.

## 2 Results and Discussion

### 2.1 Soluble Metal Ions Afford Modest Conversion of Phosphoserine and Phosphothreonine to Dehydroamino Acids

We recently reported that DHAA sites are highly abundant in protein aggregates from AD brains, leading to a variety of nucleophile‐modified residues and protein crosslinks in important brain proteins that are known to aggregate in neurodegeneration, such as Tau, CRMP2, and α‐synuclein. This observation spurred our interest in identifying an enzymatic or chemical mechanism for their formation. While seeking a human enzyme responsible for their formation, we noted the recent paper by Chandrasekar et al. describing a deoxyribozyme that produced DHAAs from phosphoserine in buffers containing ZnCl_2_, MnCl_2_, and MgCl_2_. This led us to set up an assay to screen for native ribozyme activity in RNA extracted from AD‐affected human brain tissue. Three phosphoserine‐containing peptides were incubated in the same metal‐ion‐containing buffer compositions employed by Chandrasekar et al., with or without the RNA extract. To our surprise, we detected a significant level of DHAA formation in the phosphopeptides for the negative control experiments lacking RNA. We hypothesized the metal ions present in the buffer may have been responsible for the reaction by serving as Lewis acids to facilitate phosphate β‐elimination. We followed up on these observations by evaluating the effects of a panel of divalent metal ions on a set of 7 model phosphopeptides as described in the Figure 1 legend and caption.

**FIGURE 1:**
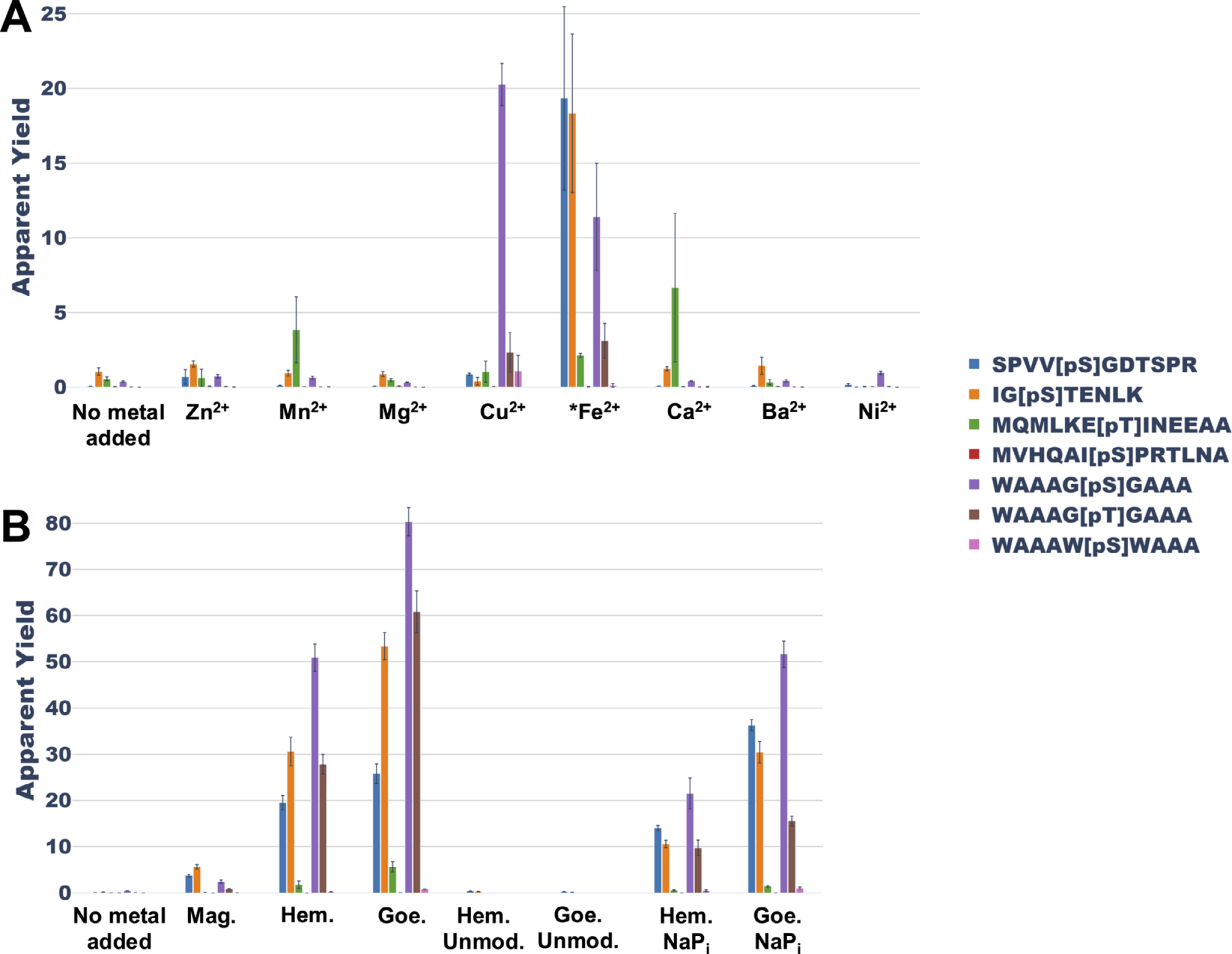
β‐elimination of phosphate at pH 7.5 from 7 model phosphopeptides catalyzed by metal species. SPVV[pS]GDTSPR and IG[pS]TENLK are phosphorylated versions of tryptic peptides including S400 and S262 of the Tau protein, and MQMLKE[pT]INEEAA and MVHQAI[pS]PRTLNA are phosphorylated versions of peptides including T72 and S16 of the HIV‐1 capsid protein. Model peptides WAAAG[pS]GAAA, WAAAG[pT]GAAA, and WAAAW[pS]WAAA were included to assess the influence of local sequence context and differences between phosphoserine and phosphothreonine reactivity. Reactions were analyzed by LC‐MS/MS, and apparent yield is defined as the intensity of the extracted ion chromatogram (XIC) for the product, divided by the summed intensities of XICs for the reactant, product, and dephosphorylated (hydrolyzed) peptide. (A) Reactions were incubated for 3 days at 37°C and contained 1 µM of each peptide (7 µM total), 50 mM HEPES (pH 7.5), 150 mM NaCl, and 1.5 mM XCl_2_, where X represents the indicated divalent cation. Reactions containing Fe^2^^+^ formed a red–orange precipitate after incubation. Data acquired at pH 8.5 are shown in Figure S1. (B) Reactions contained 1 µM of each peptide (7 µM total unless otherwise noted) in 100 mM HEPES (pH 7.5) and were incubated at 37°C for 18 h. Conditions (left to right) are as follows: no added metal (buffer control); 2% (w/v) magnetite slurry (Mag.); 2% hematite slurry (Hem.); 2% goethite slurry (Goe.); hematite slurry with unphosphorylated Tau peptides (Hem. Unmod.); goethite slurry with unphosphorylated Tau peptides (Goe. Unmod.); hematite slurry with 100 mM NaH_2_PO_4_ pH 7.5 (Hem. NaP_i_); and goethite slurry with 100 mM NaH_2_PO_4_ pH 7.5 (Goe. NaP_i_). Corresponding rates of phosphate hydrolysis for these reactions are shown in Figure S2.

Figure 1A shows the formation of DHAAs produced by a 3‐day, 37°C incubation of the 7 model phosphopeptides with 8 different divalent metal ions, each present as the dichloride salt at a concentration of 1.5 mM. All peptide identifications and reaction yield determinations were made by liquid chromatography tandem mass spectrometry (LC‐MS/MS) (see Supporting Information for details). Significant conversion (2%–20%) to DHAAs for one or more peptides was observed for Mn^2+^, Cu^2+^, Fe^2+^, and Ca^2+^, with Fe^2+^ standing out as the most effective catalyst.

We noted that in the FeCl_2_ reaction, a bright red‐orange precipitate formed, consistent with the generation of an iron hydroxide species. We therefore hypothesized that a modest increase in pH might enhance the rate of β‐elimination, potentially by increasing the availability of hydroxide for precipitation or facilitating deprotonation at the β‐position. Reactions were thus repeated at pH 8.5, with the results shown in Figure S1. Although certain metal ions, especially Zn^2^^+^, exhibited modest increases in reaction efficiency at the higher pH, overall yields remained limited.

### 2.2 Iron Oxides Catalyze Phosphate β‐Elimination With Higher Yields Than Soluble Metal Ions

In these experiments, the metal ions other than Fe^2+^ did not form a visible precipitate and exhibited much lower efficiencies than that of Fe^2+^. We therefore hypothesized that the iron‐based precipitate might act as a heterogeneous catalyst. This possibility was particularly intriguing given that iron‐based mineral deposits have been shown to accumulate in neurodegenerative disorders, including AD [15, 16, 17]. To investigate this possibility, slurries (2% w/v) of three types of insoluble iron oxides were incubated with the model phosphopeptides at pH 7.5. As shown in Figure 1B, hematite (Fe_2_O_3_), goethite (α‐FeOOH), and to a lesser extent, magnetite (Fe_3_O_4_), all promoted DHAA formation in four of the seven model phosphopeptides, including the two derived from Tau. The unphosphorylated counterparts of the two Tau‐derived peptides did not form appreciable quantities of DHAAs under these conditions (Figure 1B, Hem./Goe. Unmod.), showing that the β‐elimination reaction requires the presence of the phosphate moiety. Addition of excess phosphate (100 mM NaH_2_PO_4_ pH 7.5) did not block the reaction, although it did reduce reaction yield substantially (Figure 1B, Hem./Goe. NaPi). These results showed that heterogeneous iron oxide surfaces can promote β‐elimination in phosphopeptides under mild, near‐physiological conditions, even in biological environments containing competing ligands for the iron oxide surface.

Heterogeneous reactions have precedent in biological systems, such as when iron oxides generate reactive oxygen species through Fenton‐type chemistry [15, 18]. Heterogeneously catalyzed β‐elimination is also well established in industrial chemistry, as in the dehydration of ethanol to ethylene over Al_2_O_3_ surfaces, although such processes typically require elevated temperatures (>300°C) [19, 20]. The mineral‐assisted β‐elimination reactivity observed here occurs under much milder conditions. The conditions employed are physiologically relevant apart from the large amounts of the metal oxide catalysts (2% slurries) that were employed to expedite formation of easily measurable product quantities. Nonetheless, the same reactivity enhancements would be expected even at much lower catalyst amounts, and the DHAA‐forming reactions may thus still be important in the context of AD where the disease manifests on a timescale involving decades.

We next sought to determine the rate of iron oxide‐catalyzed phosphate β‐elimination, particularly in comparison to the spontaneous reaction, which has previously been suggested as a biochemical route for DHAA formation [13]. We measured reaction yield as a function of time for the spontaneous and iron oxide‐catalyzed reactions (Figure 2A–D). For each reaction, we employed 1 µM concentrations of each substrate phosphopeptide and, for the catalyzed reactions, a 2% slurry of the metal oxide species, providing a large excess of catalytic surface. The results were interpreted according to the Langmuir‐Hinshelwood rate law, which describes the kinetics of heterogeneous catalysis where reaction occurs to an adsorbed species on a catalyst surface, with surface reaction as the rate‐limiting step. Under the conditions employed of a vast excess of surface catalytic sites, the Langmuir‐Hinshelwood model reduces to pseudo‐first‐order behavior [21]. The data were accordingly fit to a first‐order kinetic model as described in the Methods, with fitted rate constants reported in Table S1.

**FIGURE 2:**
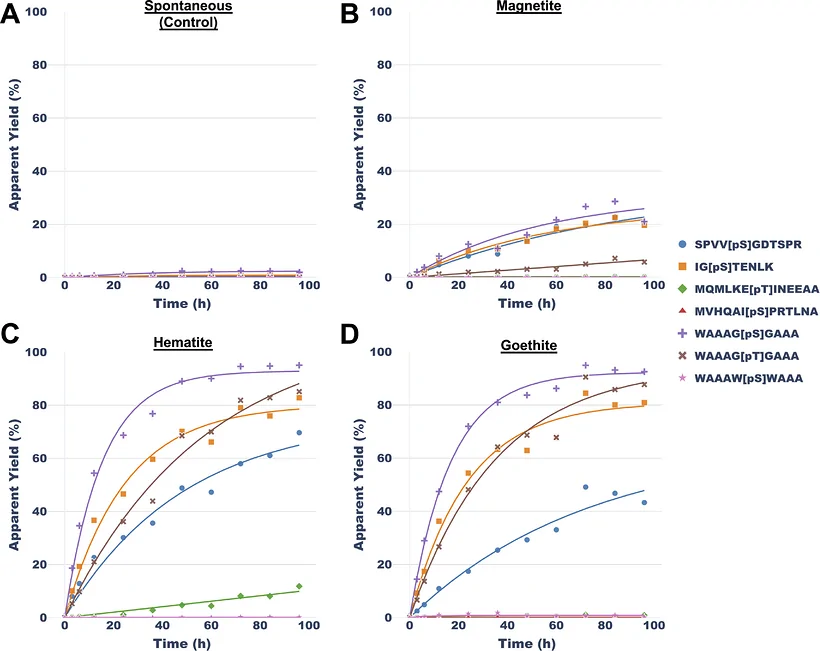
Apparent yield of DHAA formation from model phosphopeptides as a function of time. Reactions were conducted under four conditions: (A) Spontaneous reaction (negative control, no metal added), (B) 2% magnetite slurry, (C) 2% hematite slurry, and (D) 2% goethite slurry. All reactions were performed at 37°C and contained 1 µM of each peptide (7 µM total) and 100 mM HEPES (pH 7.5). Experimental data were fit to the first‐order kinetic model $Y=Y_{max}\left(1-e^{-k_{obs}t}\right)$ (see Supporting Information for details). Initial rates are given in Table 1, and the *k_obs_* and correlation coefficients determined from the fit are given in Table S1.

The initial rates of spontaneous and iron oxide‐catalyzed phosphate β‐elimination for the four most reactive peptide species (SPVV[pS]GDTSPR, IG[pS]TENLK, WAAAG[pS]GAAA, and WAAAG[pT]GAAA) are given in Table 1, along with the initial rates of the competing phosphate hydrolysis reaction. Hematite and goethite increase the rate of formation of dehydroalanine from phosphoserine by two orders of magnitude, and of formation of dehydrobutyrine from phosphothreonine by three orders of magnitude, compared to the rates of the spontaneous eliminations in the absence of added catalyst.

**TABLE 1:** Initial rate comparisons between β‐elimination of phosphate and phosphate hydrolysis for each of the tested conditions. Initial rates (*r_0_* measurements reported in percent conversion/hour) were determined from linear fits to product yields over the first 36 h of the reaction. Definition of terms: *r_0,E_—*Initial rate of β‐elimination; *r_0,E,IronOxide_*/*r_0,E,Control_—r_0,E_* for the corresponding iron oxide divided by the *r_0,E_* of the control, representing the acceleration of the reaction by a given catalyst; *r_0,H_—*Initial rate of phosphate hydrolysis; *r_0,E_*/*r_0,H_—*Initial rate of β‐elimination divided by the initial rate of phosphate hydrolysis.

Conditions	Peptide	*r_0,E_*	*r_0,E,IronOxide_*/*r_0,E,Control_*	*r_0,H_*	*r_0,E_*/*r_0,H_*
Spontaneous	SPVV[pS]GDTSPR	0.00612	—	0.0106	0.580
(Control)	IG[pS]TENLK	0.0112	—	0.00530	2.10
	WAAAG[pS]GAAA	0.0212	—	0.0819	0.259
	WAAAG[pT]GAAA	0.00150	—	0.0230	0.0650
Hematite	SPVV[pS]GDTSPR	0.933	152	0.00850	110
	IG[pS]TENLK	1.57	141	0.0124	127
	WAAAG[pS]GAAA	1.97	93.1	0.0212	92.8
	WAAAG[pT]GAAA	1.25	836	0.0127	98.4
Goethite	SPVV[pS]GDTSPR	0.698	114	0.111	6.27
	IG[pS]TENLK	1.77	158	0.0388	45.6
	WAAAG[pS]GAAA	2.20	104	0.0780	28.2
	WAAAG[pT]GAAA	1.80	1200	0.102	17.6
Magnetite	SPVV[pS]GDTSPR	0.259	42.3	0.0214	12.1
	IG[pS]TENLK	0.315	28.1	0.0607	5.19
	WAAAG[pS]GAAA	0.317	15.0	0.0849	3.74
	WAAAG[pT]GAAA	0.0656	43.9	0.0134	4.89

Goethite and magnetite both also promote hydrolysis of the phosphate moiety compared to control reactions, while interestingly hematite appeared to modestly inhibit rather than promote hydrolysis compared to control reactions. Plots of phosphate hydrolysis as a function of time are shown in Figure S3 and were used to generate the hydrolysis rates r_0,H_ in Table 1. The rate constants (*k_H_*) for the competing phosphate hydrolysis reactions are presented in Table S2. The relative effects of the catalysts on elimination vs hydrolysis of the phosphate moiety are reflected in the ratios of the initial rates for the two reactions, *r_0,E_*/*r_0,H_* (Table 1). Hematite promotes phosphate β‐elimination over phosphate hydrolysis by an average factor of ∼107 for the four phosphopeptides evaluated, with goethite at ∼24 and magnetite at ∼6.5.

### 2.3 Other Metal Oxides Also Catalyze Phosphate β‐Elimination

To assess the generality of the iron oxide catalytic activity, 2% slurries of several additional metal oxides and hydroxides were incubated with the seven model phosphopeptides, with the results shown in Figure 3. Al_2_O_3_, which is used industrially as a catalyst to convert ethanol to ethylene [19, 20], performs comparably to the iron oxides. In contrast, several other metal oxides and hydroxides, including Cu(OH)_2_, Zn(OH)_2_, and several manganese oxides, showed much less activity. TiO_2_ did not show any appreciable activity, despite it being a stronger Lewis acid than either Fe_2_O_3_ (hematite) or α‐FeOOH (goethite).

**FIGURE 3:**
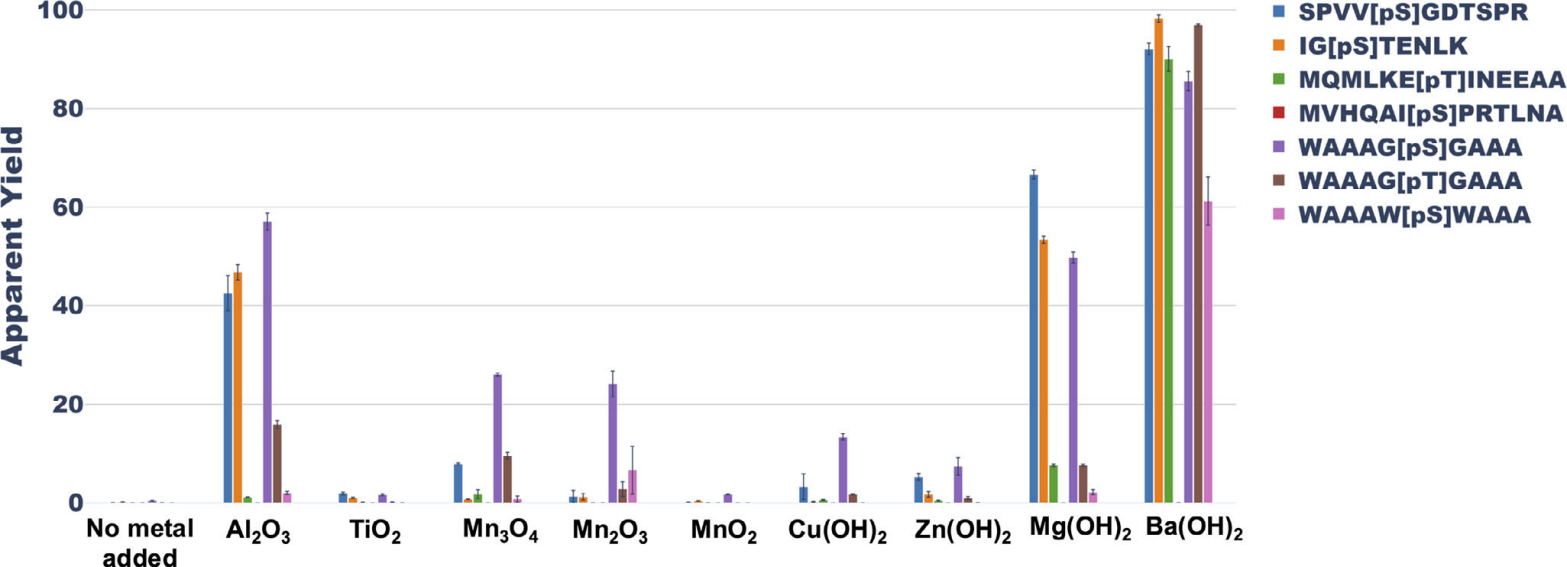
β‐elimination reactions of model phosphopeptides with metal oxides and hydroxides. All reactions contained 1 µM of each peptide (7 µM total peptide concentration) in 100 mM HEPES (pH 7.5) and were incubated for 18 h at 37°C (except positive control). All metal oxides and hydroxides were included as 2% slurries (except positive control). A positive control was provided by incubating peptides for 90 min at 37°C in 125 mM Ba(OH)_2_, which is soluble and known to catalyze β‐elimination of phosphopeptides. (4) Corresponding rates of phosphate hydrolysis for these reactions are provided in Figure S4.

Mg(OH)_2_, unlike Cu(OH)_2_ and Zn(OH)_2_, performed comparably to the iron oxides, despite Mg^2+^ being a relatively weak Lewis acid. As the most soluble compound tested, Mg(OH)_2_ raised the reaction pH to approximately 8.5, increasing the possibility that this result reflects a base‐catalyzed solution‐phase reaction rather than heterogeneous catalysis. However, we found that 1.5 mM Mg^2+^ at pH 8.5 is insufficient to drive β‐elimination (Figure S1), indicating that the reaction proceeds through a surface‐mediated mechanism. The observed activity of Mg(OH)_2_ is consistent with the well‐established role of Mg^2+^ as a phosphate‐coordinating Lewis acid in enzyme active sites [22].

We have shown here that several metal oxides and hydroxides are able to catalyze the β‐elimination of phosphate from phosphoserine and phosphothreonine in model peptides to generate DHAAs, with no elimination occurring in the absence of the phosphate group. The extension of this reaction to the modification of intact proteins, and study of the effects of the DHAA installation upon protein function are important areas for future work. The requirement for the presence of phosphate is not surprising, as phosphate is a much better leaving group than hydroxide ion [23], and the enzymes that are known to catalyze the same elimination reaction also generally require a phosphorylated substrate [3]. Phosphate's role may also involve its coordination to surface metal sites to drive binding of the phosphopeptide substrate. The elimination reaction was not strongly inhibited in the presence of excess phosphate, which was unexpected given the substantial affinities of these metal ions for inorganic phosphate [24, 25]. The absence of inhibition by phosphate supports the possibility of this reaction being operative in vivo, as otherwise the high concentration of competing surface ligands such as phosphate present in cells would likely saturate the surface binding sites.

It is interesting that while titanium oxide (TiO_2_) coordinates strongly to phosphate [26], it does not catalyze the formation of DHAAs under the conditions examined [27]. One possibility is that a multidentate interaction, likely involving both the phosphate and a carbonyl group from the amide backbone coordinated to one or more metal ion centers, helps organize the phosphopeptide into a reactive configuration, and that the necessary geometry is not afforded by the TiO_2_ surface. This is supported by our finding that, in the presence of the catalyst, elimination is greatly accelerated relative to hydrolysis. If stabilizing the phosphate leaving group was the only driving force for reaction, we would not expect a strong bias for the elimination reaction over the hydrolysis reaction. Collectively, these data are consistent with a mechanism that not only activates the phosphate leaving group, but one where the metal oxide surface stabilizes phosphopeptide conformations favorable for the geometric requirements of concerted β‐elimination. Further work will be required to fully understand the mechanism of these reactions.

### 2.4 Reaction Selectivity

The reactions of hematite, goethite, magnetite, alumina, and magnesium hydroxide with our model phosphopeptides exhibit a striking bias related to phosphopeptide sequence and structure. Four of the seven peptides consistently react efficiently, while the other three do not readily form DHAAs. The general order of reactivity is: WAAAG[pS]GAAA > SPVV[pS]GDTSPR, IG[pS]TENLK, WAAAG[pT]GAAA >> MQMLKE[pT]INEEAA >> WAAAW[pS]WAAA, MVHQAI[pS]PRTLNA. Notably, MVHQAI[pS]PRTLNA failed to form DHAAs even with Ba(OH)_2_ treatment, instead yielding the phosphate‐hydrolyzed, unmodified peptide (Figure 3). Each of the four most reactive peptides contains at least one adjacent glycine residue, suggesting that reactivity tracks with decreasing steric bulk adjacent to the phosphorylated residue. This observation is consistent with the hypothesis that multidentate coordination is a determinant of reactivity, as bulkier side chain groups may reduce the capacity of nearby carbonyls to bind the surface. Glycine is also known to disrupt peptide secondary structure due to its flexibility [28]. In peptides with secondary structure, the carbonyls are occupied in hydrogen bonding with nearby amides. Glycine, by disfavoring these intramolecular hydrogen bonds, may create a more accessible carbonyl for multidentate binding to the iron oxide surface, thus promoting the reaction.

## 3 Conclusion

DHAAs are commonly attributed to random protein damage when observed in human cells. We show here that the rate of spontaneous formation of DHAAs from phosphoserine and phosphothreonine residues is appreciable, but slow (Figure 2A). This elimination reaction directly competes with the alternative pathway of phosphate hydrolysis, which yields an unmodified serine or threonine residue and thus a normal and undamaged protein. The balance that normally exists between these two reaction pathways, whereby one path leads to an irreversibly modified and reactive form of the protein, and the other to an undamaged protein, could be perturbed by the presence of metal oxides, that preferentially drive elimination over hydrolysis. Such a disruption of normal proteostasis could play a role in the induction of protein aggregation processes.

Our proteomic analyses on brain protein aggregates revealed increases of up to two orders of magnitude in abundance for particular DHAA sites from AD brains compared to the age‐matched control brains [11]. This observation suggests that DHAA formation may be condition‐dependent and potentially driven by a biochemical factor that is accentuated in AD. Metal oxides have notably been shown to accumulate in AD [15, 16, 17]. Iron oxides, in particular, accumulate within the cortex and hippocampus (regions most severely affected by AD) [29, 30] and the extent of iron deposition correlates with cognitive decline [16, 31]. Iron deposits are also commonly found within or nearby the hallmark pathogenic aggregates in AD: amyloid‐β plaques and Tau tangles [32, 33, 34]. DHAAs have been shown to be prevalent in the pathogenic, aggregates of Tau protein [11]. Tau is an intrinsically disordered protein that largely lacks secondary structure and is natively hyperphosphorylated as an early step in AD pathogenesis [29], making it a plausible substrate for the metal‐oxide‐catalyzed β‐elimination, consistent with the results presented here for elimination of phosphate from model Tau phosphopeptides. Future work will be necessary to determine whether or not DHAAs are generated in vivo by this mechanism, to explore further the reaction mechanism and substrate scope, and to understand what role this reaction may play in protein aggregation processes and neurodegenerative disease.

## Author Contributions

**Samuel W. Markovich**: conceptualization, investigation, writing – original draft, methodology, validation, visualization, writing – review and editing, software, formal analysis, data curation. **Brian L. Frey**: conceptualization, writing – review and editing, methodology, validation, formal analysis, funding acquisition, supervision. **Mark Scalf**: investigation, methodology, funding acquisition. **Lloyd M. Smith**: conceptualization, funding acquisition, writing – review and editing, methodology, supervision.

## Conflicts of Interest

The authors declare no conflicts of interest.

## Supporting information



The authors have cited additional references within the Supporting Information [35, 36].**Supporting File 1**: anie74402‐sup‐0001‐SuppMat.docx.

**Supporting File 2**: anie74402‐sup‐0002‐SuppMat.xlsx.

**Supporting File 3**: anie74402‐sup‐0003‐SuppMat.txt.

## Data Availability

The data that supports the findings of this study are available in the Supporting File of this article.
