# Supplementary material for: Metal Oxides Catalyze Formation of Dehydroamino Acids by Phosphate β‐Elimination Under Physiological Conditions
Source: Angew Chem Int Ed Engl. 2026 Sep 1;65(42):e9805066. doi: 10.1002/anie.9805066 (PMC13577452; doi:10.1002/anie.9805066)
Supplement: Supplementary file 1 [file ANIE-65-e9805066-s001.docx]

**Materials and Methods**

**Reaction of phosphopeptides with metal oxides**

Phosphopeptides were purchased from Thermo Scientific and stored as 1-2 mg/mL stock solutions in water at -20ºC. All reactions were performed in triplicate unless otherwise stated. Prior to each experiment, a mixed peptide stock containing 50 μM of each peptide was prepared from the individual frozen peptide stocks.

For soluble ion reactions (Figure 1A, Supplementary Figure 1), each 100 μL reaction was generated in a 1.5 mL Protein LoBind tube (Eppendorf) by first adding 78 μL water, 10 μL 1.0 M HEPES pH 7.5/8.5 (depending on reaction), and 10 μL of a 15 mM solution of the metal chloride salt. Then, 2 μL of the 50 μM peptide stock solution was added at the end to initiate the reaction. Samples were briefly vortexed and centrifuged to collect the solution at the bottom of the tube. Reactions were incubated for 72 hours at 37 ºC. In the FeCl_2_ condition, the precipitate was pelleted at 20,000xg for 2 minutes at room temperature and the supernatant was collected. EDTA was added to a final concentration of 15 mM and samples were diluted to 150 μL.

For reactions with metal oxides, a 10% (w/v) slurry was prepared for each metal oxide. Each slurry was bath sonicated for 10 minutes to break up any agglomerates. All metal oxides were purchased from Sigma-Aldrich.

For single time point reactions, a reaction mixture was generated in a 2mL Protein LoBind tube by first adding 68 μL water, 10 μL 1.0 M HEPES pH 7.5, and 2 μL of the 50 μM peptide stock solution. Reactions were initiated by addition of 20 μL of the appropriate 10% slurry. Samples were briefly vortexed and centrifuged to collect the solution at the bottom of the tube. Reactions were incubated in a Thermo Scientific Thermomixer at 37 ºC, 1000 rpm. After 18 h, 100 μL of 200 mM NaH_2_PO_4_ pH 7.5, 400 mM EDTA was added to each reaction. The resulting solution was vortexed vigorously and centrifuged at 20,000xg for 2 minutes at room temperature. 150 μL of supernatant was transferred to a new, 1.5 mL Protein LoBind tube.

For kinetic measurements, a single large-volume reaction was prepared using the same final concentrations as above. To a 2 mL Protein LoBind tube, 952 μL water, 140 μL 1.0 M HEPES pH 7.5, and 28 μL of the 50 μM peptide stock solution was added. To initiate the reaction, 280 μL of water (spontaneous reaction, negative control condition), or of a 10% slurry of magnetite, hematite, or goethite was added. Samples were briefly vortexed and placed in a benchtop centrifuge to concentrate the solution at the bottom of the tube. Reactions were incubated in a Thermo Scientific Thermomixer at 37 ºC, 1000 rpm, as before. Aliquots were taken at 3, 6, 12, 24, 36, 48, 60, 72, 84, and 96 h. For each time point, a homogeneous mixture was created by several rounds of end-over-end mixing. 100 μL of the reaction was aliquoted from the tube and was added to 100 μL of 200 mM NaH_2_PO_4_ pH 7.5, 400 mM EDTA. The reaction mixtures were briefly vortexed and centrifuged to collect the solution at the bottom of the tube and placed back on the Thermomixer. The aliquot was vortexed vigorously and centrifuged at 20,000xg for 2 minutes at room temperature. 150 μL of supernatant was transferred to a new, 1.5 mL Protein LoBind tube.

**Sample Preparation for LC-MS/MS**

Each 150 μL sample was acidified with 3 μL of 20% trifluoracetic acid (TFA) for a final concentration of 0.4%. Samples were immediately desalted using a C18 solid-phase extraction pipette tip (Pierce™ C18 Pipette Tips, 100 μL, Thermo Fisher Scientific) according to manufacturer’s protocols. Desalted samples were dried using a SpeedVac Concentrator and reconstituted in 20 μL of 5% acetonitrile (ACN) with 0.2% formic acid to prepare them for analysis by liquid chromatography-tandem mass spectrometry (LC-MS/MS).

**LC-MS/MS analysis of model phosphopeptides**

Samples were analyzed by HPLC-MS/MS using a system consisting of an HPLC (nanoAcquity, Waters) connected to an electrospray ionization (ESI) Orbitrap mass spectrometer (QE-HF, ThermoFisher Scientific). HPLC separation employed a 365 μm diameter fused silica capillary micro-column packed with 20 cm of 1.7 μm-diameter, 130 Angstrom-pore-size, C18 beads (Waters BEH), with an emitter tip pulled to approximately 1 μm using a laser puller (Sutter instruments). Peptides were loaded on-column at a flowrate of 400 nL/min for 30 min and then eluted over 30 min for model peptides at a flowrate of 300 nL/min with a gradient of 5% to 35% acetonitrile, in 0.1% formic acid. Full-mass profile scans were performed in the FT orbitrap between 375-1500 m/z at a resolution of 120,000, followed by MS/MS HCD scans of the ten highest intensity parent ions at 30% relative collision energy and 15,000 resolution, with a mass range starting at 100 m/z. Dynamic exclusion was enabled with a repeat count of one over a duration of 30 seconds.

**Data analysis**

Each .raw file was analyzed in QualBrowser. Extracted ion chromatograms (XICs) with 0.02 m/z windows were generated to represent the most abundant m/z species for each peptide measured, as determined using the negative control and Ba(OH)_2_ positive control. The species were validated using corresponding fragmentation information using MetaMorpheus search software.^[35]^ For each of the seven model peptides, XICs for the phosphopeptide, DHAA-containing peptide, and unmodified peptide were generated, resulting in 21 total XICs per spectrum. The total peak area representing the peptide species in the XIC was measured using the built-in Qualbrowser integration function. This was recorded as the intensity of each species.

The apparent yield of the DHAA-containing peptide was calculated by dividing the intensity of that peptide (product) by the summed intensities of the DHAA-containing peptide, phosphorylated peptide, and unmodified peptide. Similarly, the apparent yield of phosphate hydrolysis was calculated by dividing the intensity of the unmodified peptide by the same denominator. The apparent yield for the triplicate reactions was averaged and plotted with error bars representing the standard error of each calculation.

Kinetic data for the rate of β-elimination were fit to a first order kinetic model, defined by equation 1.

$Y=Y_{max}(1-e^{-k_{obs}t})$	(1)

Y represents reaction yield and k_obs_ represents the observed rate constant that represents a parameter encompassing contributions from adsorption and the rate of surface reaction.^[21]^ Y_max_ represents the empirically determined maximum yield, the value to which some of the fits asymptotically approach. Parameters were fit to the model using nonlinear least-squares regression using SciPy.^[36]^ Initial parameter estimates were generated based on the observed maximum signal and empirical scaling of the data.

Kinetic data for the initial rate of β-elimination and phosphate hydrolysis were fit to a linear model, as shown in equation 2.

$Y=k_{obs}t$	(2)

**Supplementary Figure 1:** β-elimination of phosphate at pH 8.5 from 7 model phosphopeptides catalyzed by metal species. Reactions were analyzed by LC-MS/MS and apparent yield is defined as the intensity of the extracted ion chromatogram (XIC) for the product, divided by the summed intensities of XICs for the reactant, product, and dephosphorylated (hydrolyzed) peptide. Reactions were incubated for 3 days at 37 °C and contained 1 µM of each peptide (7 µM total), 50 mM HEPES (pH 8.5), 150 mM NaCl, and 1.5 mM XCl₂, where X represents the indicated divalent cation. Reactions containing Fe²⁺ formed a red–orange precipitate after incubation. As noted in the Figure 1 caption, the 7 peptides were chosen as follows: SPVV[pS]GDTSPR and IG[pS]TENLK are phosphorylated versions of tryptic peptides including S400 and S262 of the Tau protein, and MQMLKE[pT]INEEAA and MVHQAI[pS]PRTLNA are phosphorylated versions of peptides including T72 and S16 of the HIV-1 capsid protein. Model peptides WAAAG[pS]GAAA, WAAAG[pT]GAAA, and WAAAW[pS]WAAA were included to assess the influence of local sequence context and differences between phosphoserine and phosphothreonine reactivity.

**Supplementary Figure 2:** The apparent yield of phosphate hydrolysis at pH 7.5 is reported for each reaction. This figure complements Figure 1B, which plots elimination of phosphopeptides. Reactions contained 1 µM of each peptide (7 µM total unless otherwise noted) in 100 mM HEPES (pH 7.5) and were incubated for 18 h. Conditions (left to right) are as follows: no added metal (buffer control); 2% (w/v) magnetite slurry (Mag.); 2% hematite slurry (Hem.); 2% goethite slurry (Goe.); hematite slurry with 100 mM NaH₂PO₄ pH 7.5 (Hem. NaP_i_); and goethite slurry with 100 mM NaH₂PO₄ pH 7.5 (Goe. NaP_i_).

**Supplementary Table 1**: Kinetic parameters and initial rate comparisons between β-elimination of phosphate and phosphate hydrolysis for each of the tested conditions. Initial rates (*r_0_* measurements reported in percent conversion/hour) were determined from linear fits to product yields over the first 36 hours of the reaction. Definition of terms: *k_obs_* – rate constant determined by a first-order fit to the β-elimination reaction using equation 1; *r_0,E_* – initial rate of β-elimination; R^2^*_E_* – correlation coefficient for the linear fitting to the initial rate of β-elimination; *r_0,E,IronOxide_* / *r_0,E,Control_* – *r_0,E_* for the corresponding iron oxide divided by the *r_0,E_* of the control, representing the acceleration of the reaction with a given catalyst; *r_0,H_* – initial rate of phosphate hydrolysis; R^2^*_H_* – correlation coefficient for the linear fitting to the initial rate of phosphate hydrolysis; *r_0,E_* / *r_0,H_* – initial rate of β-elimination divided by the initial rate of phosphate hydrolysis.

| Condition | Peptide | *k_E_* (h^-1^) | *r_0,E_* | R^2^*_E_* | *r_0,E,IronOxide_* / *r_0,E,Control_* | *r_0,H_* | R^2^*_H_* | *r_0,E_* / *r_0,H_* |
| --- | --- | --- | --- | --- | --- | --- | --- | --- |
| Spontaneous  (Control) | SPVV[pS]GDTSPR | - | 0.00612 | 0.993 | - | 0.0106 | 0.987 | 0.58 |
|  | IG[pS]TENLK | - | 0.0112 | 0.944 | - | 0.0053 | 0.434 | 2.1 |
|  | WAAAG[pS]GAAA | - | 0.0212 | 0.983 | - | 0.0819 | 0.966 | 0.259 |
|  | WAAAG[pT]GAAA | - | 0.0015 | 0.961 | - | 0.023 | 0.994 | 0.065 |
| Hematite | SPVV[pS]GDTSPR | 0.0205 | 0.933 | 0.908 | 152 | 0.0085 | 0.98 | 110 |
|  | IG[pS]TENLK | 0.0404 | 1.57 | 0.926 | 141 | 0.0124 | 0.703 | 127 |
|  | WAAAG[pS]GAAA | 0.0650 | 1.97 | 0.853 | 93.1 | 0.0212 | 0.298 | 92.8 |
|  | WAAAG[pT]GAAA | 0.0176 | 1.25 | 0.97 | 836 | 0.0127 | 0.925 | 98.4 |
| Goethite | SPVV[pS]GDTSPR | 0.0137 | 0.698 | 0.991 | 114 | 0.111 | 0.995 | 6.27 |
|  | IG[pS]TENLK | 0.0423 | 1.77 | 0.939 | 158 | 0.0388 | 0.244 | 45.6 |
|  | WAAAG[pS]GAAA | 0.0598 | 2.2 | 0.922 | 104 | 0.078 | 0.499 | 28.2 |
|  | WAAAG[pT]GAAA | 0.0282 | 1.8 | 0.991 | 1200 | 0.102 | 0.991 | 17.6 |
| Magnetite | SPVV[pS]GDTSPR | 0.0111 | 0.259 | 0.937 | 42.3 | 0.0214 | 0.981 | 12.1 |
|  | IG[pS]TENLK | 0.0178 | 0.315 | 0.923 | 28.1 | 0.0607 | 0.936 | 5.19 |
|  | WAAAG[pS]GAAA | 0.0183 | 0.317 | 0.813 | 15 | 0.0849 | 0.767 | 3.74 |
|  | WAAAG[pT]GAAA | - | 0.0656 | 0.931 | 43.9 | 0.0134 | 0.875 | 4.89 |

**Supplementary Figure 3:** Apparent yield of peptide phosphate hydrolysis as a function of time. Reactions were conducted under four conditions: **A)** Spontaneous reaction (negative control, buffer only, no metal added). **B)** 2% magnetite slurry, **C)** 2% hematite slurry, and **D)** 2% goethite slurry. Experimental data were fit with a modified, integrated first order kinetic model. All reactions were performed at 37 °C and contained 1 µM of each peptide (7 µM total) and 100 mM HEPES (pH 7.5). Experimental data were fitted using a first-order kinetic model. This figure serves as a complement to Figure 2, in that the data were obtained from the same reactions and LC-MS/MS analyses. The corresponding rate constants (*k_H_*) determined from the fit are reported in Supplementary Table 2.

**Supplementary Table 2**: Rate constants determined by zero-order kinetic (linear) fitting for peptide phosphate hydrolysis under the four conditions tested. Goethite generally promoted phosphate hydrolysis to a greater extent than hematite or magnetite, whereas hematite appreciably accelerated hydrolysis only for MVHQAI[pS]PRTLNA, a peptide not conducive to phosphate elimination (Figure 3). Correlation coefficients (R²) for the linear fits used to estimate rate constants are provided. In several cases, hydrolysis was minimal, and linear fitting was therefore not considered reliable (R² < ~0.6).

| Condition | Peptide | *k_H_* (μM/h) | R^2^ |
| --- | --- | --- | --- |
| Control | SPVV[pS]GDTSPR | 0.0258 | 0.835 |
|  | IG[pS]TENLK | 0.00786 | 0.888 |
|  | MQMLKE[pT]INEEAA | -0.00119 | 0.0886 |
|  | MVHQAI[pS]PRTLNA | -0.00058 | 0.389 |
|  | WAAAG[pS]GAAA | 0.109 | 0.975 |
|  | WAAAG[pT]GAAA | 0.0367 | 0.982 |
|  | WAAAW[pS]WAAA | 0.00421 | 0.849 |
| Hematite | SPVV[pS]GDTSPR | 0.00876 | 0.959 |
|  | IG[pS]TENLK | 0.00427 | 0.569 |
|  | MQMLKE[pT]INEEAA | 0.00967 | 0.848 |
|  | MVHQAI[pS]PRTLNA | 0.0869 | 0.978 |
|  | WAAAG[pS]GAAA | -0.0014 | 0.0111 |
|  | WAAAG[pT]GAAA | 0.00977 | 0.863 |
|  | WAAAW[pS]WAAA | 0.00657 | 0.363 |
| Goethite | SPVV[pS]GDTSPR | 0.117 | 0.991 |
|  | IG[pS]TENLK | 0.0404 | 0.761 |
|  | MQMLKE[pT]INEEAA | 0.0560 | 0.957 |
|  | MVHQAI[pS]PRTLNA | 0.00632 | 0.567 |
|  | WAAAG[pS]GAAA | 0.0325 | 0.508 |
|  | WAAAG[pT]GAAA | 0.0630 | 0.930 |
|  | WAAAW[pS]WAAA | 0.0114 | 0.461 |
| Magnetite | SPVV[pS]GDTSPR | 0.0117 | 0.922 |
|  | IG[pS]TENLK | 0.00288 | 0.0123 |
|  | MQMLKE[pT]INEEAA | 1.88e-05 | 0.00048 |
|  | MVHQAI[pS]PRTLNA | 0.000139 | 0.0260 |
|  | WAAAG[pS]GAAA | 0.0954 | 0.846 |
|  | WAAAG[pT]GAAA | 0.0188 | 0.906 |
|  | WAAAW[pS]WAAA | 0.00196 | 0.126 |

**Supplementary Figure 4:** Phosphate hydrolysis for reactions of model phosphopeptides with metal oxides and hydroxides. All reactions contained 1 µM of each test peptide (7 µM total peptide concentration) in 100 mM HEPES (pH 7.5) and were incubated for 18 h at 37C (except positive control). All metal oxides and hydroxides were included as 2% slurries (except positive control). A positive control was provided by incubating peptides for 90 min at 37C in 125 mM Ba(OH)₂, which is soluble and known to catalyze β-elimination of phosphopeptides. This figure serves as a complement to Figure 3 in that the data were obtained from the same reactions and LC-MS/MS analyses.

**References**

[21]	K. V. Kumar, K. Porkodi, F. Rocha, “Langmuir–Hinshelwood kinetics – A theoretical study” *Catalysis Communications* **2008**, *9*, 82–84.

[30]	R. M. Miller, R. J. Millikin, Z. Rolfs, M. R. Shortreed, L. M. Smith in *Statistical Analysis of Proteomic Data* (Ed.: T. Burger), Springer US, New York, NY, **2023**, pp. 35–66.

[31]	P. Virtanen, R. Gommers, T. E. Oliphant, M. Haberland, T. Reddy, D. Cournapeau, E. Burovski, P. Peterson, W. Weckesser, J. Bright, S. J. Van Der Walt, M. Brett, J. Wilson, K. J. Millman, N. Mayorov, A. R. J. Nelson, E. Jones, R. Kern, E. Larson, C. J. Carey, İ. Polat, Y. Feng, E. W. Moore, J. VanderPlas, D. Laxalde, J. Perktold, R. Cimrman, I. Henriksen, E. A. Quintero, C. R. Harris, A. M. Archibald, A. H. Ribeiro, F. Pedregosa, P. Van Mulbregt, SciPy 1.0 Contributors, A. Vijaykumar, A. P. Bardelli, A. Rothberg, A. Hilboll, A. Kloeckner, A. Scopatz, A. Lee, A. Rokem, C. N. Woods, C. Fulton, C. Masson, C. Häggström, C. Fitzgerald, D. A. Nicholson, D. R. Hagen, D. V. Pasechnik, E. Olivetti, E. Martin, E. Wieser, F. Silva, F. Lenders, F. Wilhelm, G. Young, G. A. Price, G.-L. Ingold, G. E. Allen, G. R. Lee, H. Audren, I. Probst, J. P. Dietrich, J. Silterra, J. T. Webber, J. Slavič, J. Nothman, J. Buchner, J. Kulick, J. L. Schönberger, J. V. De Miranda Cardoso, J. Reimer, J. Harrington, J. L. C. Rodríguez, J. Nunez-Iglesias, J. Kuczynski, K. Tritz, M. Thoma, M. Newville, M. Kümmerer, M. Bolingbroke, M. Tartre, M. Pak, N. J. Smith, N. Nowaczyk, N. Shebanov, O. Pavlyk, P. A. Brodtkorb, P. Lee, R. T. McGibbon, R. Feldbauer, S. Lewis, S. Tygier, S. Sievert, S. Vigna, S. Peterson, S. More, T. Pudlik, T. Oshima, T. J. Pingel, T. P. Robitaille, T. Spura, T. R. Jones, T. Cera, T. Leslie, T. Zito, T. Krauss, U. Upadhyay, Y. O. Halchenko, Y. Vázquez-Baeza, “SciPy 1.0: fundamental algorithms for scientific computing in Python” *Nat Methods* **2020**, *17*, 261–272.
